# A Systematic Review on the Effects of Psychosocial Interventions on Quality of Life and Functioning Outcomes Among Populations Experiencing Ongoing Threat

**DOI:** 10.1002/cpp.70262

**Published:** 2026-03-28

**Authors:** See Heng Yim, Apostolos Polymerou

**Affiliations:** ^1^ Department of Psychology University of Hong Kong Hong Kong Special Administrative Region China; ^2^ London Metropolitan University London UK

**Keywords:** conflict, humanitarian, psychological trauma, quality of life, systematic review, violence

## Abstract

Humanitarian emergencies increasingly involve ongoing traumatic stress, characterised by a lack of environmental safety that is often required for recovery. Evaluating quality of life (QoL) and functioning is essential in these contexts, as symptom reduction alone may not capture the multidimensional nature of resilience and social adaptation under continuous threat. This systematic review aims to evaluate the effectiveness of psychosocial interventions on nonsymptom‐focused outcomes—specifically QoL and functioning—for populations facing ongoing violence or interpersonal violence. Following PRISMA guidelines, a systematic search of PubMed, Medline and PsycINFO was conducted. Interventions were categorised using layers two to four of the International Red Cross and Red Crescent Movement's MHPSS framework. Data from 18 studies (17 unique studies) comprising 3165 participants were synthesised using a narrative approach. Evidence indicates that both trauma‐ and present‐focused interventions were likely to improve QoL and functioning despite ongoing threat. Comparison between interventions remained inconclusive. There were also discrepancies between informants regarding intervention outcomes. Future research should include operationalisation of ‘ongoing threat’ where possible and include and standardise nonsymptom‐focused outcomes when evaluating interventions.

## Summary

- Through this systematic review, practitioners can recognise that both trauma‐ and present‐focused psychosocial interventions are effective in improving quality of life (QoL) and daily functioning, even when populations remain under ongoing threat. However, evidence remains inconclusive as to which therapeutic framework is superior, as few studies conducted direct comparisons between the two frameworks. Practitioners are encouraged to adopt a ‘both/and’ therapeutic stance—validating the realistic nature of ongoing risks while simultaneously helping clients differentiate between past traumas and present dangers.
- The review shows that task sharing is a feasible and successful delivery model in low‐resource or conflict‐affected areas, although regular supervision needs to be in place particularly in lower resource settings.
- Future research should prioritise standardising nonsymptom‐focused outcomes, such as QoL and functioning, and provide explicit documentation of implementation fidelity and supervision structures.

## 1 Introduction

The world faces rising humanitarian emergencies, with increased human‐induced crises such as industrial accidents and conflicts, alongside climate‐related natural hazards (Ager et al. 2022). In 2024, 61 state‐based conflicts in 36 countries marked the highest since 1946 (Rustad 2025). Emergencies are categorised into sudden‐onset, slow‐onset and protracted crises (World Health Organization 2013). Populations in conflict or instability often endure significant mental health challenges (Charlson et al. 2019). Researchers examining continuous exposure to traumatic stress have raised scepticism about the term posttraumatic stress disorder (PTSD), which may not accurately reflect the realities of people experiencing continuous traumatic stress (CTS) (Nuttman‐Shwartz and Shoval‐Zuckerman 2016) or ongoing traumatic stress (OTS) (Diamond et al. 2010). CTS/OTS may have a different symptom profile, with hypervigilance seen as adaptive in ongoing threat contexts (Eagle and Kaminer 2013). Individuals may not re‐experience trauma but instead anticipate future threats (Somer and Ataria 2015). Prior trauma reduces coping capacity to subsequent stressors (Breslau et al. 2008), and the trauma index remains contested in classification (McNally 2003; Weathers and Keane 2007). These insights emphasise the need to study CTS separately from PTSD or CPTSD. Although the idea of Criterion A index trauma is subject to nosological controversy (McNally 2003; Weathers and Keane 2007), these insights emphasise the need to study CTS separately from PTSD or CPTSD.

Psychological and psychosocial interventions for this population vary widely, from community activities to specialised therapies, with differing effectiveness (Purgato et al. 2018). Many are designed for postconflict settings and may not suit ongoing conflicts. Interventions designed to address these challenges have historically been shaped by two dominant frameworks: trauma‐focused and psychosocial approaches (Miller and Rasmussen 2024). The debate between these approaches centres on the primary drivers of distress in war‐affected communities, with trauma‐focused advocates emphasising the direct impact of war exposure and psychosocial perspectives highlighting the mediating role of daily stressors (Miller and Rasmussen 2010). Although both frameworks offer valuable insights, a critical gap remains in how intervention effectiveness is evaluated. Current research frequently prioritises symptom reduction, particularly decreases in PTSD and depression, as the primary outcome measure. This emphasis is evident in meta‐analyses examining the efficacy of psychotherapies for PTSD in ongoing conflict settings, which often focus on symptom severity as the key indicator of success (Yim et al. 2024).

This narrow focus neglects the broader impact of interventions on overall quality of life (QoL) and functioning and how symptom relief enhances QoL. QoL, a key aspect of health, is defined by the WHO as individuals' perception of their position in life within their cultural and value context, linked to their goals and expectations (Kuyken et al. 1995). Functioning closely relates to QoL determinants (Leventhal and Colman 1997). QoL is recognised as multidimensional, encompassing physical wellbeing, social relationships, community engagement, recreation, personal development and fulfilment (Flanagan 1978). In war‐affected populations, QoL is influenced by PTSD‐related impairments and social changes such as increased poverty, reduced employment, shifting social networks and environmental factors (Tol et al. 2010). Research in the Balkans shows both PTSD and postwar social conditions independently impact QoL (Matanov et al. 2013). To enhance treatment for populations under ongoing threat, focusing on functioning and QoL is crucial. Addressing social determinants through community and systemic interventions can also influence health‐seeking behaviours in conflict zones (Munezero and Manoukian 2021).

Meta‐analyses demonstrate that psychotherapies, particularly cognitive‐behavioural therapy (CBT), can improve QoL in civilian populations with PTSD (Fortin et al. 2021; Kaur et al. 2024). Kaur et al. (2024) conducted a meta‐analysis on the effectiveness of exposure‐based therapies for improving QoL in individuals with PTSD. The study found that from the 20 studies included, exposure‐based therapies were significantly more effective than control conditions at posttreatment, with a medium effect size. However, their effect was small compared to nontrauma‐focused therapies. At follow‐up (ranging from 1 month to 2 years), exposure therapies showed negligible effects on QoL. Authors suggested that rather than the effectiveness of exposure therapies being subdued with time, they argued that the nonsignificant effect could be due to the higher efficacy of comparison treatments used in studies that had a follow‐up period. Additional analyses of specific QoL domains revealed no significant differences. Fortin et al. (2021) conducted a meta‐analysis of effects of psychotherapies on PTSD symptoms among civilian populations. Of the nine studies included, they noted similar findings in CBT, with medium effects compared to waitlist‐controlled studies and small effects compared to nontrauma‐focused interventions. Theses reviews, however, did not specifically focus on ongoing threat.

This systematic review aimed to extend the previous review conducted by Yim et al. (2024) on symptom‐focused effects of psychological interventions under ongoing threat, by examining the effectiveness of psychosocial interventions on nonsymptom‐focused outcomes, specifically QoL and functioning. This review extended previous findings to include non‐RCTs and populations where the stressors and trauma are not over. By synthesising existing evidence, this review seeks to provide a more holistic understanding of intervention effectiveness and inform the development of interventions that focus on the wider determinants of health. Furthermore, this review will contribute to the debate between trauma‐focused and psychosocial approaches by demonstrating the importance of considering both symptom reduction and QoL outcomes when evaluating intervention success, particularly in contexts of continued threat.

### 1.1 Research Questions

What is the effectiveness of psychosocial interventions on nonsymptom‐focused outcomes, particularly quality of life (QoL) and functioning, in the context of ongoing threats, namely, ongoing armed conflicts and interpersonal violence? A secondary aim is to examine the characteristics of these interventions, including their adaptations to the ongoing threat context and to the local culture.

## 2 Method

### 2.1 Protocol Registration

This review was conducted and reported in accordance with the Preferred Reporting Item for Systematic Reviews and Meta‐Analysis (PRISMA) standards (Page et al. 2021). The protocol was preregistered on PROSPERO (CRD420251047219).

### 2.2 Definitions

#### 2.2.1 Ongoing Threat

To avoid confusion, we did not use the term CTS due to varied definitions regarding whether ongoing stress includes daily stressors (Nuttman‐Shwartz and Shoval‐Zuckerman 2016). We also avoided the term ‘ongoing traumatic stress reaction’, common in Middle Eastern conflicts (Hamadeh et al. 2025). While introducing new terminology has its drawbacks, we use ‘ongoing threat’ to prevent misunderstandings within this review's context. Following Yim et al. (2024), we defined a traumatic event per Diagnostic and Statistical Manual of Mental Disorders (DSM‐5) definition of a traumatic event as an actual or threatened risk of death or physical harm experienced, witnessed or learned about (American Psychiatric Association and American Psychiatric Association 2013). ‘Ongoing threat’ refers to individuals in precarious, dangerous situations, including sociopolitical conflicts caused by organised violence, and intimate or interpersonal violence (IPV). It encompasses repeated exposure to violence, abuse, attacks, bombings, killings or similar ongoing threats. The index trauma is defined as the worst single incident or multiple closely related incidents causing distress, as per the Clinician‐Administered PTSD Scale for DSM‐5 (Weathers et al. 2018) as either the worst single incident (e.g., ‘the accident’) or multiple but closely related incidents that caused distress (e.g., multiple bombings and repeated IPV). Unlike Yim et al. (2024), refugees, we also include refugees, internally displaced persons, and asylum seekers if the articles justify ongoing, current threats similar to the index trauma, recognising that many still live in precarious conditions (Miller and Rasmussen 2024).

#### 2.2.2 Classifying Psychosocial Interventions

The classification of the psychosocial interventions was based on the International Red Cross and Red Crescent Movement's Mental Health and Psychosocial Support Framework (The International Red Cross and Red Crescent Movement (RCRC), n.d). The framework represents the framework of MHPSS that are required to address the needs of individuals, families and communities. The level of supervision, skills and competencies increases with the layers, with Level 1 includes basic psychosocial support (universal), Level 2 includes focused psychosocial support for people at risk (e.g., structured activities for children and youth and peer support and group work), Level 3 includes psychological support and Level 4 include specialised mental health care. The International RCRC movement framework was chosen because terminologies such as wellbeing and psychological distress are used over mental health disorders.

#### 2.2.3 Population, Intervention, Comparison, Outcomes and Study Design (PICOS)

Table 1 lists out the PICOS of the review.

**TABLE 1:** PICOS.

	Inclusion criteria	Exclusion criteria
Population	All adult, child and adolescent samples experiencing ongoing threat.	Combat/veteran/military‐related trauma or medical trauma; postconflict areas (unless there is evidence that ongoing conflict/threat/unrest persists), refugees resettled in camps in another country that is not experiencing conflicts, developmental trauma or adverse childhood experiences, complex trauma, children affected by parental conflict intervention(s)
Intervention	Psychological, psychosocial and psychologically informed intervention across level two to four of the International Movement RCRC framework (The International Red Cross and Red Crescent Movement (RCRC), n.d). These include focused psychosocial support, psychosocial support and specialised mental health care.	Level 1 of the Movement Framework (basic psychosocial support).
Outcomes	Measures related to wellbeing and functioning such as quality of life, functioning, coping, resilience, wellbeing and resilience.	
Setting	Any setting.	
Study design	Randomised controlled trials, controlled and uncontrolled studies. Quantitative studies. Mixed‐method studies.	Studies reporting secondary data. Qualitative studies. Book chapters, dissertations, conference abstracts. Descriptive case studies with no mention of outcomes. Non–peer‐reviewed articles. Editorials or opinion pieces. Reviews, grey literature, non‐English language.

#### 2.2.4 Search Strategy

The search strategy included database search and citation search. The keywords were chosen with reference to Yim et al. (2024). The search included title and abstract search alongside MeSH terms search. Search terms were variations of (1) trauma/stress, (2) violence and conflict, (3) psychological interventions and (4) quality of life/wellbeing. PubMed, Medline and PsycINFO were searched. The detailed search terms are listed in Supporting Information. Citation search was mainly taken from Yim et al. (2024) on studies where quality of life was measured. The database search was conducted on 13 January 2025, and citation search was conducted between March and July 2025.

#### 2.2.5 Study Selection

Duplicated papers were initially detected automatically and resolved manually on Rayyan (Ouzzani et al. 2016) by the first author. Preliminary screening of the titles and abstract was carried out by two authors (SHY and AP) independently, with inconsistencies (marked as ‘Maybes’ on the software) resolved through discussion. Full texts of the remaining papers were then downloaded. SHY and AP screened roughly 20% of the studies independently and the remaining studies were screened by one person. Inconsistencies were resolved through discussion. The main areas of inconsistencies included the definition of ongoing threat as well as whether the outcomes were nonsymptom‐focused.

#### 2.2.6 Data Extraction and Analysis

The setting, design, participant characteristics, how ongoing threats were conceptualised, intervention details and outcome measures relating to the review question (e.g., quality of life and resilience) were extracted by the second author. Cultural adaptations were extracted following an existing cultural adaptation framework (Day et al. 2023). Following that, a narrative synthesis approach (Davis et al. 2009) was adopted to analyse and summarise the extracted data by SHY and AP. This process involved summarising key findings in tables, comparing the results across studies, identifying, synthesising and critiquing the study findings.

#### 2.2.7 Use of Artificial Intelligence Tools

The articles were uploaded to Elicit AI for information extraction, after which all outputs were manually reviewed to ensure accuracy. Elicit AI demonstrated high precision in identifying relevant information and was user‐friendly, facilitating efficient cross‐checking against the original articles. However, it missed some statistical details in tables and those had to be added manually, and the provided reasoning was relatively shallow and lacked qualitative nuance.

#### 2.2.8 Quality Appraisal

As this review is intended to include randomised controlled and nonrandomised, cross‐sectional studies, the CASP checklists (Critical Appraisal Skills Programme (CASP) 2024) were used according to the study type (e.g., CASP for cross‐sectional studies and for randomised controlled trials [RCTs]). The two authors independently assessed 20% of the included papers, and disagreements between the two authors over the risk of bias in particular studies were discussed and resolved. They then split the remaining papers and separately assessed. Strengths, weaknesses or unknowns were reported in Table S1.

Overall, studies were strong at explaining the significance of the research question and the constraints under realistic, low‐resource, conflict‐ridden settings. However, the cross‐sectional studies had notable limitations, including lack of power calculations, the use of non‐locally validated questionnaires, the omission of information about fidelity and the failure to report effect sizes (Farchi and Gidron 2010). Mpande et al. (2013) noted that the reliability estimates for the measures were low and studies had small sample sizes. For RCTs, risks of bias included not including information about blinding (in particular investigator and assessor blinding given it is not possible to include blind participants in psychological interventions), unequal baseline characteristics across groups such as perceived stress (Orang et al. 2018), symptoms (Bass et al. 2013), which could potentially influence the outcome. Reporting the precision of treatment effects was poor in some studies such as reporting only *p* values without effect sizes (e.g., Dinmohammadi et al. 2021) or confidence intervals (e.g., Orang et al. 2018).

## 3 Results

**FIGURE 1:**
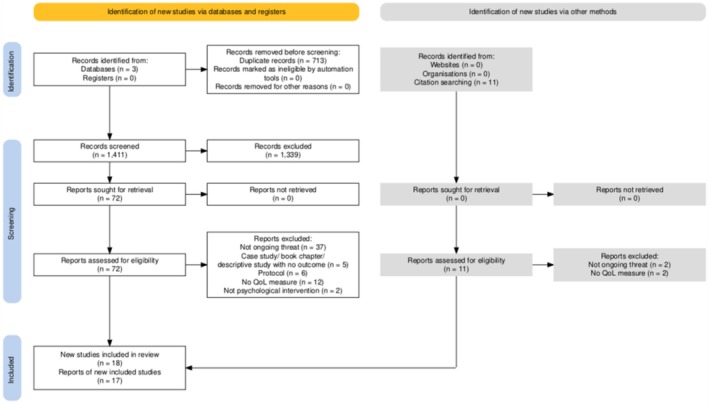
PRISMA flowchart.

A total of 18 records that include 17 unique studies were included, resulting in a total of 3165 participants. The PRISMA flowchart (Figure 1) (Haddaway et al. 2022) details the number of papers omitted and included at each stage of the process.

### 3.1 Characteristics of Included Studies

Table S2 outlines study settings, the level of development of the regions as indicated by the World Bank at the time of the study, design and participant characteristics. The 18 studies were across 10 locations—Israel (*n* = 5), Iraq (*n* = 3), DRC (*n* = 1), Zimbabwe (*n* = 1), Uganda (*n* = 2), Iran (*n* = 2), Nepal (*n* = 1), Lebanon (*n* = 1), Sri Lanka (*n* = 1) and Indonesia (*n* = 1)—South America was unrepresented.

Ten studies adopted randomised controlled trials (RCT) (Bass et al. 2013; Dinmohammadi et al. 2021; Farchi and Gidron 2010; Knaevelsrud et al. 2015; Orang et al. 2018; Puvimanasinghe and Price 2016), in which two were cluster RCT (Jordans et al. 2010; Tol et al. 2010) and one was a pilot RCT (Miller et al. 2020). Five studies used a quasiexperimental design (Ager et al. 2011; Berger et al. 2007, 2012; Gelkopf and Berger 2009; Mpande et al. 2013). Three studies lacked controls (Abdulah and Abdulla 2019; Bass et al. 2022; Wagner et al. 2012).

Two studies explored ongoing interpersonal and sexual violence (Dinmohammadi et al. 2021; Orang et al. 2018) (both were conducted in Iran). Two unique studies explored sexual violence in the context of other adversity and armed conflicts (Bass et al. 2013, 2022) in the DRC and in Iraq (Abdulah and Abdulla 2019). All other studies were related to ongoing community violence or armed conflicts. Ten studies focused on adults, and eight studies targeted children and adolescents.

In terms of gender, studies on sexual violence/interpersonal violence focused exclusively on females (Abdulah and Abdulla 2019; Bass et al. 2013; Orang et al. 2018; Dinmohammadi et al. 2021). Other studies had a skewed ratio of predominantly female participants (Farchi and Gidron 2010; Knaevelsrud et al. 2015; Wagner et al. 2012). One study exclusively focused on male participants (Gelkopf and Berger 2009), and two studies had more male than female participants (Mpande et al. 2013; Puvimanasinghe and Price 2016). For studies that had a balanced gender ratio, gender‐specific findings were noted—Tol et al. (2008) showed a significant effect of gender in reducing functioning impairment in girls but not boys.

### 3.2 Conceptualisation of Ongoing Threat

Table S3 lists how ongoing threat was defined or interpreted across the included studies. None of the studies operationalise ongoing threat, and they were all descriptive. For interpersonal violence, ongoing threat includes living with the abusive partner (Orang et al. 2018; Dinmohammadi et al. 2021), multiple ongoing threats including staying in the same place as the site of sexual violence and unstable regions with armed groups' activity, fighting and robbery (Bass et al. 2013), survivors of rape, abduction and being sold who now lived in refugee camps and were under realistic threat of the precariousness of the camp conditions and the risk of being found by the armed groups (Abdulah and Abdulla 2019). For armed conflicts and community violence, ongoing threat included (1) direct conflict exposure: ongoing armed conflicts and war, for example, an explicit mention of daily missile attacks, and (2) ongoing stressors and instability: where the researchers mentioned the war has ended, ongoing threat was conceptualised by the experience of persisting ongoing stressors, violence and instability. For example, Ager et al. (2011) described the aftermath of armed conflict with widespread social problems such as abduction and violence; Jordans et al. (2010) mentioned ongoing political violence after the civil war; precarious living conditions and instability in refugee camps (Miller et al. 2020); and (3) targeted violence: continuous threats of reprisal, abduction or torture were described (Mpande et al. 2013; Puvimanasinghe and Price 2016; Tol et al. 2008).

### 3.3 Intervention Details

#### 3.3.1 Qualifications of the Interventionists

Table S4 outlines intervention details, delivery modes and adaptations. Most utilised task‐sharing with trained lay facilitators (teachers, research assistants and psychosocial aides), except five involving specialists (internet CBT: Wagner et al. 2012; Knaevelsrud et al. 2015; solution‐focused counselling: Dinmohammadi et al. 2021; and narrative exposure therapy: Orang et al. 2018; ventilation and psychological inoculation: Farchi and Gidron 2010). The majority of interventions were delivered in face‐to‐face group settings; three studies utilised distant delivery modes including telephone counselling and internet trauma‐focused CBT. Sessions typically lasted 60–90 min and spanned 5 weeks to 4 months, although long‐term telephone counselling averaged 36.2 sessions.

#### 3.3.2 Group Interventions

Group‐based interventions were the most widely used modality. Six studies adopted school‐based group programmes targeting children and adolescents. The classroom programmes incorporated eclectic approaches that included creative expression, CBT principles (15‐session Psychosocial Structured Activities, PSSA: Ager et al. 2011; 12‐session ERASE‐Stress intervention: e.g., Gelkopf and Berger 2009), as well as voluntary narrative exposure through drawing (15‐session Classroom‐based intervention, CBT: Tol et al. 2008; Jordans et al. 2010). For nonclassroom‐based group interventions, Abdulah and Abdulla (2019) examined a 2‐month art‐based programme (four 4‐h sessions per week) with an art instructor. For group‐based interventions for adults, two interventions specifically focused on trauma. Group Cognitive Processing Therapy (CPT) consisted of one individual and 11 group sessions delivered by two psychosocial assistants (Bass et al. 2013). For a bottom‐up approach, a 3‐day community, Tree of Life Trauma Healing Workshop (TOL) that focused on connecting with others and discussing traumatic experiences was examined (Mpande et al. 2013).

For interventions that did not specifically focus on trauma, 16‐session group Interpersonal Psychotherapy (IPT‐G) interventions serving specific psychosocial goals (Bolton et al. 2007) were delivered by lay facilitators. A nine‐session Caregiver Support Intervention (CSI) included stress and relaxation, positive parenting, anger management, coping with stress and adversity and was delivered (Miller et al. 2020).

#### 3.3.3 Individual Interventions

For individual nontrauma‐focused treatments, six‐session solution‐focused counselling was delivered for pregnant women experiencing intimate partner violence (Dinmohammadi et al. 2021).

Trauma‐focused interventions included testimonial therapy where narration of traumatic experience was involved (Puvimanasinghe and Price 2016); 10‐session web‐based CBT (Wagner et al. 2012; Knaevelsrud et al. 2015); and narrative exposure therapy (Orang et al. 2018).

#### 3.3.4 Levels of the Interventions According to the RCRC Movement Framework

Based on the RCRC Movement's MHPSS framework, the interventions detailed in Table S2 are categorised into Focused Psychosocial Support and Psychological Support. None of the included studies were classified as Specialised Mental Health Care.

##### 3.3.4.1 Level 2: Focused Psychosocial Support

This level involves focused support for individuals, families or groups at risk, typically involving manualised activities or psychosocial support designed to promote positive mental health, for example, Psychosocial Structured Activities (PSSA): a school‐based programme in Uganda (Ager et al. 2011), classroom‐based interventions on stress (e.g., Gelkopf and Berger 2009; Jordans et al. 2010; Tol et al. 2008), Art‐Based Groups (Abdulah and Abdulla 2019), Caregiver Support Intervention (CSI) (Miller et al. 2020), creative play (Bolton et al. 2007) and group IPT (Bolton et al. 2007). Some interventions were delivered as task‐sharing interventions but involved a higher level of expertise such as group CPT (Bass et al. 2013) where trauma narration was involved.

##### 3.3.4.2 Level 3: Psychological Support

This level involves more specialised skills and includes trauma processing, typically delivered by professionals, such as NET (Orang et al. 2018), testimonial therapy (Puvimanasingh & Price, 2016), web‐based trauma‐focused CBT (Wagner et al. 2012) and solution‐focused counselling (Dinmohammadi et al. 2021).

#### 3.3.5 Supervision

Details on supervision are listed in Table S4. Recording of supervision was inconsistent in terms of frequency and supervision format. In those where supervision was recorded, a multitiered supervision system was common, often relying on international experts in lower resource settings. For example, in the DRC, psychosocial assistants had weekly telephone or in‐person meetings with local supervisors, who were then supervised by international clinical social workers (Bass et al. 2013). In Uganda, local staff with IPT‐G experience provided weekly supervision, overseen by US‐based trainers (Bolton et al. 2007). The Caregiver Support Intervention (CSI) in Lebanon employed a structured hierarchy where nonmental health specialists received three on‐site observations and weekly supervision from a trained social worker, who was in turn supervised by a doctoral‐level psychologist.

In a high‐income setting (e.g., Israel), the classroom‐based interventions were delivered by teachers and supervision was recorded. Supervision involved three 3‐h sessions in Berger et al. (2007), three 90‐min sessions in Gelkopf and Berger (2009) and six 2‐h group sessions in Berger et al. (2012). Direct observations of sessions took place to ensure fidelity. Another intervention conducted in Israel did not report any information on supervision/fidelity measures (Farchi and Gidron 2010).

Conversely, some focused psychosocial programmes in lower resource settings, such as the PSSA programme in Uganda, reported that supervision was limited due to significant resource constraints, despite teachers receiving initial training (Ager et al. 2011). Abdulah and Abdulla (2019) also reported that there was a lack of standardised training of art therapists in Iraq, which could impact the quality of the art‐based group intervention. Others did not report supervision/fidelity (e.g., Puvimanasinghe and Price 2016; Dinmohammadi et al. 2021; Jordans et al. 2010).

### 3.4 Cultural Adaptations

Using the coding framework by Day et al. (2023), studies discussed several tailoring, cultural adaptations or modifications due to ongoing threat, which were synthesised below and described in Table S4. In contrast, several interventions did not report any tailoring or modifications (Abdulah and Abdulla 2019; Ager et al. 2011; Berger et al. 2007; Dinmohammadi et al. 2021; Farchi and Gidron 2010; Gelkopf and Berger 2009; Jordans et al. 2010). However, some of those were delivered by local therapists which might mean that adaptations occurred naturally. Another intervention was developed locally and hence cultural adaptations might not be required (e.g., Mpande et al. 2013).

#### 3.4.1 Content Tailoring/Refining

Several interventions were modified to incorporate cultural or religious practices such as religious stories and meditative prayers (Gelkopf and Berger 2009), Koranic quotes and metaphors (Knaevelsrud et al. 2015; Wagner et al. 2012).

Language was a common modification. Internet‐based Cognitive‐Behavioural Treatment (CBT) manuals were translated into Arabic (Wagner et al. 2012) or Sri Lankan language (Puvimanasinghe and Price 2016). Group Cognitive Processing Therapy (CPT) manuals were adapted and translated, and content was modified to fit Congolese culture (Bass et al. 2013). The language complexity was reduced by adding pictorial illustrations to fit the literacy level.

Culturally, interventions adopted a more directive therapeutic stance, respecting cultural norms by discouraging disclosure of sexual violence to family members due to potential consequences (Knaevelsrud et al. 2015; Wagner et al. 2012) and addressing beliefs about sexual assault such as beliefs relating to social status (Bass et al. 2013).

#### 3.4.2 Relationship Fidelity

Two interventions described discussing with the local populations regarding the intervention acceptability, aligning the components with the participants' cultural views. For example, for IPT‐G, prior qualitative work in Uganda indicated that the emphasis on group relationship building was culturally compatible (Bolton et al. 2007), and the CSI in Lebanon went through local feedback and revision and were facilitated by same‐gender facilitators to fit with the cultural norms (Miller et al. 2020).

#### 3.4.3 Context Adaptations (Format and Setting)

For accessibility needs in resource‐constraint settings, for example, in the study in Northern Iraq, art‐based sessions were held within the Internally Displaced Persons (IDP) Camp to facilitate attendance (Abdulah and Abdulla 2019). For CPT in the DRC, psychosocial assistants sometimes organised meetings in forests/fields during displacement to maintain therapy access during periods of insecurity (Bass et al. 2013). To adapt to the ongoing threat context, interventions addressed safety concerns at the beginning and were organised with flexible logistics and keeping participation secret from families (Orang et al. 2018). Interventions also added content relating to affect modulation, self‐affirmations and social skills due to the ongoing threat (Gelkopf and Berger 2009).

### 3.5 Outcomes

Outcome measures, findings and implementation challenges are listed in Table S5. Studies used standardised scales as well as locally developed scales. For standardised scales, the Flourishing Scale (FS), Short Form Health Survey (SF‐36) and EUROHIS‐QOL were used. Locally derived scales included Zimbabwe Community Life Questionnaire (ZCLQ) and Sri Lanka Index of Psychosocial Status (SLIPSS‐A). Study‐specific scales included Functional impairment scale based on 20 important tasks of daily living (Bass et al. 2013, 2022) and gender‐specific scales for youth in camps (Bolton et al. 2007).

#### 3.5.1 Uncontrolled Studies

Studies without a control group generally reported larger effect sizes and positive findings. In Iraq, an uncontrolled pilot study with a small sample of web‐based trauma‐focused CBT for PTSD reported a large increase in quality of life (*d* = 1.17) (Wagner et al. 2012). However, caution needs to be exercised as the study sample size was small due to security and privacy concerns from the participants where the final sample might be highly selective. Similarly, an art‐based intervention in the same region observed a very large improvement in psychological flourishing (*d* = 1.89) among a small sample of 14 participants (Abdulah and Abdulla 2019). Bass et al. (2022) conducted a long‐term follow‐up study on the effect of CPT and found that the reduction in functional impairment was maintained across 6‐month, 18‐month and 5‐year follow‐up periods. However, they only followed up with people allocated to CPT instead of the control condition.

#### 3.5.2 Waitlist Control (WLC)/Treatment as Usual

Interventions compared against a waiting list control consistently demonstrated statistically significant improvements in the treatment group. Multiple studies evaluating school‐based CBT‐informed programme conducted in Israel found significant improvements in functional impairment compared to waitlist controls, with one study indicating medium effect sizes (e.g., *η* ^2^ = 0.12) (Berger et al. 2012), but no effect size was reported in others (Berger et al. 2007; Gelkopf and Berger 2009). In Iraq, an RCT of web‐based trauma‐focused CBT reported a significant increase in life satisfaction compared to waitlist control, with a moderate *within‐group* effect size (*d* = 0.76) (Knaevelsrud et al. 2015). In Nepal, the intervention group showed a significantly greater reduction in functional impairment compared to the waitlist control (*d* = 0.58) (Jordans et al. 2010). In Sri Lanka, testimonial therapy significantly improved general psychosocial functioning using a locally developed scale for rural Sri Lanka over time compared to controls, though it failed to produce significant changes in the well‐being scores on the WHO‐5 index (Puvimanasinghe and Price 2016).

#### 3.5.3 Active Control

Comparison with active controls yielded a more nuanced picture. CPT significantly reduced functional impairment compared to individual support (*d* = 1.1) in a study in DRC (Bass et al. 2013). However, the between‐group long‐term effect was unknown as the authors only followed up with the participants in the CPT arm (Bass et al. 2022). In Zimbabwe, both ‘Tree of Life’ and psycho‐education programmes revealed moderate increases in community engagement and attitudes towards community healing (*d* = 0.39–0.57). Nevertheless, the two groups were statistically comparable across most wellbeing outcomes (Mpande et al. 2013). In Iran, although participants in both NET and Treatment‐as‐Usual (TAU) showed significant within‐group improvements in functioning, there was no significant between‐group difference at the 3‐ or 6‐month follow‐up (Orang et al. 2018). Similarly, in Uganda, participants in IPT‐G reported greater reductions in functional impairment than those in creative play or control groups, but these differences did not reach statistical significance (Bolton et al. 2007). In Israel, a psychological inoculation intervention showed no significant difference in daily functioning when compared to a ventilation group (Farchi and Gidron 2010).

#### 3.5.4 Individual Differences in Outcomes

Several studies frequently highlighted informant discrepancies. In Lebanon, the caregiver support intervention significantly outperformed the waitlist control in caregiver well‐being (*d* = 0.43) and parent‐reported child well‐being (*d* = 0.51), yet the child‐reported outcomes remained nonsignificant compared to the control group (Miller et al. 2020). Similarly, in Indonesia, child‐rated functioning significantly improved compared to the WLC (*d* = 0.42), but parent‐rated measures showed no significant difference (Tol et al. 2008). In Uganda, although children and parents reported significant wellbeing outcomes, this was not corroborated by teachers (Ager et al. 2011). Some studies also found gender differences; in Israel, boys showed a significantly larger reduction in functional impairment compared to girls (Berger et al. 2007). Conversely, in Indonesia, the intervention was found to be effective in reducing functional impairment for girls but showed no significant effect among boys (Tol et al. 2008).

## 4 Discussion

The review sought to examine the effectiveness of psychosocial interventions on nonsymptom focused outcomes, particularly QoL and functioning in the context of ongoing threat, namely, ongoing violence and interpersonal violence. As a secondary question, the characteristics of the interventions, including their adaptations to the ongoing threat context and to the local culture, were also reviewed. This study is an extension of Yim et al. (2024) review where only PTSD‐related outcomes were reviewed. The review included 17 unique studies across 10 geographical locations. The theoretical frameworks of the interventions appear to be primarily shaped by trauma‐focused, present‐focused, psychosocial and ecological frameworks. The trauma‐focused framework included the processing of traumatic memories using interventions such as cognitive processing therapy (CPT), narrative exposure therapy (NET), trauma‐focused CBT and testimony therapy. The present‐focused approaches included solution‐focused counselling and interpersonal therapy (IPT). Other approaches encompassed broader psychosocial considerations, such as caregiver support interventions, tree‐of‐life circles and the Psychosocial Structured Activities Programme (PSSA), where community support and links were emphasised. The synthesis of findings indicates that both trauma‐ and present‐focused interventions are likely to be effective in improving QoL and daily functioning.

Whether trauma‐focused therapy or present‐focused therapy yielded better nonsymptom‐focused outcomes were inconclusive, as no study directly compared these two modalities. Although some studies focused on comparing an intervention with an active control, the interventions might not be fully fitted into either trauma‐ or present‐focused category especially for integrative interventions. In addition, the results were more mixed when the interventions were compared to an active control. For example, IPT‐G in Uganda (Bolton et al. 2007), tree‐of‐life intervention in Zimbabwe (Mpande et al. 2013) or narrative exposure therapy in Iraq (Orang et al. 2018) did not reach statistical significance when compared to controls. Due to the heterogeneity of the interventions, direct comparisons were not possible, particularly when it was across different layers of the RCRC framework (The International Red Cross and Red Crescent Movement (RCRC), n.d) with varying levels of intensity. Some interventions were more preventative (e.g., school‐based interventions), whereas others were more focused on trauma (e.g., CPT).

This review affirms that psychotherapies can improve QoL in individuals with PTSD, even amidst ongoing threats (Fortin et al. 2021; Kaur et al. 2024). However, the longevity of these effects remains uncertain due to scarce long‐term follow‐up studies. Postintervention environments influence natural recovery, especially where conditions change unpredictably (Hamadeh et al. 2025). Discrepancies between child, parent and teacher reports highlight the need for triangulating stakeholder data, particularly in youth research.

Integrating this review with Yim et al. (2024) offers a broader perspective on trauma symptoms, QoL and nontrauma impacts. Findings suggest interventions are feasible and effective, even when delivered by paraprofessionals and amid active threats. Few studies overlapped in the two reviews (*n* = 6). This indicates a research gap regarding both symptom alleviation and overall life functioning. Unlike Yim et al. (2024), we did not distinguish types of ongoing violence due to their frequent co‐occurrence—such as gender‐based and political violence—especially affecting women, complicating efforts to distinctly categorise and analyse each form of violence as a potential moderator. This review extends Yim et al.'s work by incorporating cultural adaptation frameworks (Day et al. 2023) and included more studies on ongoing threat. In addition to task‐sharing, content must be culturally tailored—translations, spiritual practices and local metaphors—and delivered flexibly—sessions in camps or remote locations—to ensure relevance. Relationship fidelity through community feedback enhances cultural congruence. Despite promising results, many studies faced limitations such as small sample sizes, lack of power calculations and high attrition (e.g., due to people being displaced and return migration; Ager et al. 2011), hampering long‐term conclusions.

### 4.1 Limitations

The systematic review reveals that although interventions can yield sustained positive outcomes amidst ongoing threat, there are several methodological challenges and quality issues that could impact the transferability to advance the field. Many regions where there were active conflicts at the time of writing, such as Ukraine, Burkina Faso, Sudan and Gaza, were not included in the review following our inclusion/exclusion criteria. In the current review, 4 studies took place in high‐income settings at the time of the studies, 3 in upper‐middle‐income countries and 11 in lower middle‐ or low‐income countries. Resource implications can be different where an emergency occurs in a high‐resource versus a low‐resource setting. For example, lower resource settings require training of lay workers to deliver task‐sharing interventions and regular supervision due to the lack of qualified mental health professionals. It is also unknown how many of these interventions are being routinely implemented in the settings beyond the research studies.

The primary methodological weakness observed across the sources is the pervasive challenge of maintaining rigour in study design in unstable settings. Although RCTs were used in half of the included studies, the quality appraisal process reveals that some studies suffered from a lack of control groups without spillover effects (e.g., Abdulah and Abdulla 2019; Mpande et al. 2013; Wagner et al. 2012), rendering it difficult to isolate the specific intervention effects. Another concern was the sample size. Many studies were noted as being underpowered or failed to report power calculations (e.g., Bolton et al. 2007; Farchi and Gidron 2010; Knaevelsrud et al. 2015), undermining confidence the interpretations of the findings.

Several measures used were criticised for having unsatisfactory internal reliability in the specific population (e.g., Jordans et al. 2010; Tol et al. 2008) or for lacking local validation. The heterogeneity of measures used and locally derived scales make it difficult to do meta‐analysis on the findings to understand the pooled effects.

### 4.2 Clinical Implications

#### 4.2.1 Supervision

Based on the included studies, task‐sharing interventions were common in ongoing threat settings. The shortage of specialists probably necessitates shifting what would normally be more specialised interventions in high‐resource settings (such as CPT and IPT) to be delivered by paraprofessionals. To maintain quality and safety, this ‘task shifting’ requires upfront training and continuous, multitiered supervision networks connecting local workers to higher level clinical experts, including remote supervision from higher resource settings. However, the intensity of supervision was highly dependent on the setting's resources and logistical challenges (e.g., travel restrictions and security incidents). In a low‐income context (e.g., Uganda), supervision was limited (Ager et al. 2011). Conversely, in high‐income contexts (e.g., Israel), supervision was structured; teachers received multiple 3‐h or 90‐min supervisory sessions with the programme trainers or manual authors, and trainers directly observed the classroom sessions to ensure adherence (e.g., Berger et al. 2007, 2012). Future studies should clearly document supervision, and interventions will need to include ongoing supervision in the planning.

#### 4.2.2 Ongoing Threat

Although the evidence regarding trauma‐ and present‐focused interventions is inconclusive and previous reviews did not find any harm in trauma‐focused work in ongoing threat settings (e.g., Yim et al. 2024), others cautioned that processing in the ‘classic PTSD’ way or reducing ‘hypervigilance’ might not be the predominant goal when the individual is in survival mode and when attending to threats is adaptive (Pressley et al. 2025). Safeguarding and ‘do no harm’ principles need to be upheld. As past research in the Balkans and other conflict zones shows that postwar social conditions and PTSD symptoms independently impact QoL (Matanov et al. 2013), in ongoing threat settings, a ‘both/and’ approach is probably also needed—validating the reality of ongoing risk while concurrently helping the client differentiate between past traumas and present threats. Clinicians need to avoid retraumatisation and iatrogenic harm. Some adaptations might include limited processing (e.g., processing specific intrusive memories that directly impact on adaptive functioning; Dieste and Gottlieb 2025), ongoing consent at the beginning of each session for trauma‐focused work while attending to power imbalances (Orang et al. 2018; Pressley et al. 2025), ensuring cultural relevance, as well as safety planning to distinguish between past trauma and present danger (Dieste and Gottlieb 2025). Rather than choosing between trauma‐ and present‐focused models, clinicians are recommended to also consider functioning‐based outcomes, for example, the ability of an individual to perform daily tasks and maintain social connections to survive in a continuous traumatic environment. In this respect, interventions are advised to incorporate ecological aspects and integrate psychosocial perspectives to enhance community connections and maximise the use of the local resources.

### 4.3 Research Implications

To improve operationalisation and assist researchers in assessing intervention applicability, Table 2 summarises our recommendations for future research. In contexts where randomisation was used, baseline differences between intervention and control groups were frequently noted (e.g., Bass et al. 2013; Dinmohammadi et al. 2021; Jordans et al. 2010), potentially limiting the comparability of results. Baseline differences, particularly gender disparities, should be addressed to refine interventions. Many conflict‐affected areas remain unstudied, especially regarding nonsymptom outcomes.

**TABLE 2:** Recommendations for future studies.

Domain	Description
I. Operationalising threat	
1. Definition of Ongoing Threat	Clear, specific description of the ongoing threat (e.g., domestic violence, political violence and economic insecurity) beyond historical conflict, include questionnaires that also address ongoing threat in addition to posttraumatic stress.
2. Measurement of contextual insecurity	Description of how setting‐level insecurity was objectively or subjectively measured during the trial period (e.g., incident reports and participant perceptions)
II. Feasibility and ethics	
3. Documentation of challenges	Explicit reporting of logistical challenges (travel restrictions, technical issues and privacy issues) and security incidents that impacted delivery.
4. Justification for design	Clear justification for the chosen design (e.g., quasiexperimental over RCT) based on political, ethical or practical needs.
III. Implementation fidelity	
5. Fidelity documentation	Use of structured checklists, observation or other methods to assess adherence and competency of facilitators, including documentation of supervision structures.
IV. Outcome measurement	
6. Local validation	Reporting of the psychometric properties of all measures specific to the target population and language.
7. Triangulation of data	Efforts to use multi‐informant reports (child, parent and teacher) for studies on youth.
8. Nonsymptom focused outcome	Where appropriate, include nonsymptom focused outcomes such as quality of life and wellbeing based on what is important to the community and population studied.

Due to increased task‐sharing in MHPSS, the RCRC framework requires clearer classification criteria for intervention layers, including precise definitions and consideration of professional qualifications. For instance, a low‐intensity intervention by a qualified psychologist may be higher in the hierarchy than a trauma‐focused intervention by lay counsellors. The intervention content within focused psychological support also varies greatly. For example, a classroom intervention involving trauma narration (e.g., Tol et al. 2008; Jordans et al. 2010) could be considered more intensive than a classroom intervention on stress and present‐focused strategies (e.g., Berger et al. 2012). A group IPT intervention might also require different levels of supervision compared to a classroom‐based intervention. We anticipate that other experts might disagree with how the interventions were classified in this review. We recommend refining the classifications to improve future programme planning.

In the current body of work, consistent documentation and definition of the context of ongoing threat is lacking. Several studies were excluded as the researchers only mentioned ‘conflict affected’ rather than whether the conflict was ongoing or passed. In areas of conflicts, multiple types of violence often co‐occur. Studies should better measure the psychosocial needs of vulnerable groups—women, children, disabled and elderly—and detail how insecurity affects study feasibility (e.g., logistical challenges such as travel restrictions, difficulty securing parental consent and the inability to trace participants who migrated; privacy and trust in the research team and resource constraints), implementation and outcomes. Building trust through local partnerships and documenting intervention fidelity are vital.

Finally, harmonising nonsymptom outcome measures and creating a common outcome dataset with validated tools is essential to produce reliable, comparable evidence that captures outcomes meaningful to affected populations.

## Funding

The authors have nothing to report.

## Ethics Statement

The authors have nothing to report.

## Conflicts of Interest

The authors declare no conflicts of interest.

## Supporting information


**Table S1:** Quality Appraisal.
**Table S2:** Study Characteristics.
**Table S3:** Conceptualisations of Ongoing Threat.
**Table S4:** Intervention details.
**Table S5:** Outcomes and Implementation Challenges/Considerations.

## Data Availability

No primary data were included. All the tables and data generated from the synthesis are provided in the Supporting Information.
