# Supplementary material for: A Systematic Review on the Effects of Psychosocial Interventions on Quality of Life and Functioning Outcomes Among Populations Experiencing Ongoing Threat
Source: Clin Psychol Psychother. 2026 Mar 28;33(2):e70262. doi: 10.1002/cpp.70262 (PMC13032185; doi:10.1002/cpp.70262)
Supplement: Supplementary file 1 — Table S1: Quality Appraisal. Table S2: Study Characteristics. Table S3: Conceptualisations of Ongoing Threat. Table S4: Intervention details. Table S5: Outcomes and Implementation Challenges/Considerations. [file CPP-33-e70262-s001.docx]

**Supplementary Information Table S1. Quality Appraisal**

| **Cross sectional studies** | Issue focused | Appropriateness of method | Subject recruitment | Accuracy of measures | Data collection | Participant number | Result presentation | Rigor of data analysis | Statement of findings | Can the result be applied | Value of the research | Positives, Negatives or Unknowns |
| --- | --- | --- | --- | --- | --- | --- | --- | --- | --- | --- | --- | --- |
| Wagner et al. (2012) | Yes | Yes. Taking into account that it is a pilot study. | Yes. | Yes | Yes | No. Few males (2 males), small sample size (15). | Effect sizes and clinically significant improvements presented. | Can’t tell. Unknown fidelity of the therapist. | Yes - no confidence interval; p value and effect size reported | Yes | Valuable as there is few research on internet treatments for ongoing threat. | Negatives: Lack of a randomized control group. |
| Mpande et al. (2013) | Yes | Yes. Authors acknowledged that a randomised controlled design would not be possible in the small community setting. | Yes | Can't tell. The SRQ results need to be interpreted with caution as there was a low reliability estimate of .492 (Cronbach’s alpha).  Only 25 people took part in the validation. | Yes. | Yes. | Yes. | Can't tell. They did not describe the tree of life intervention in detailed enough. There were more common elements across conditions than the researchers had planned. Possibility of therapist drift. | Yes - p values and effect sizes reported. | Yes - fits the population of ongoing insecurity | Yes - good efforts in community-driven approach | Strengths of this study include samples sizes that provide sufficient statistical power, low experimental mortality, comparable naturalistic settings, the use of blind assessors, the use of a measure of psychological health that is reasonable reliable to the local community. nature of safety in interventions conducted within a context of ongoing violence.  Limitations and justification: There’s no waitlist control condition for comparison. RCT was not possible because the tight-knit communities would contaminate the treatments through sustained discussion, interaction, and possible responses about being in different interventions. Decided that creating a known validity threat (that is, non-equivalent groups) that could be explicitly examined in analysis and interpretation was better than creating a known validity threat that could not be so examined (that is, contamination of treatments). |
| Ager et al (2011) | Yes | Can’t tell. Not a full RCT and tossing a coin may be biased. | Yes | Can't tell. Brief ethnographic interviewing, idiosyncratic scale rather than psychometrics, subjective and not validated | Yes. Determining local, contextual and culturally-grounded understanding of wellbeing | Yes. But there is no power calculation | No. Included teacher, parent, child findings - *p* values presented, no effect size (idiosyncratic measure) | Yes | Yes | Yes | Can’t tell. From the design of the current study it is not possible to say what components of the PSSA intervention were most active. | Strengths: Good to include drop-out analysis - those retained were significantly older and had higher self-report well-being scores at baseline.  Limitations: over 150 children were not available at the time of follow-up data collection |
| Abdullah & Abdulla, 2019 | Yes | Can’t tell. Key research question about effectiveness of the intervention cannot be answered with this study design | Yes – biases were accounted for | Can't tell. Psychometric properties of the measures were not reported in this sample; the authors reported the general properties of the measure | Yes | Can't tell. No power calculation | Yes | Can’t tell. Due to lack of power calculation | Can't tell. The design of the study does not allow this. | Yes | Yes | Limitations: Small size and unclear power calculation for the t-test. |
| Farchi & Gidron, 2010 | Yes | Yes | Yes | Yes | Yes | Can't Tell | Can't Tell | Yes | Yes | Can't Tell | Yes | Positives: Clear research question in a challenging setting  Negatives/ Unknowns:  Unclear about power calculation, no effect sizes |
| Puvimanasinghe & Price, 2016 | Yes | Yes | Yes | Yes | Yes | Don't Know | Can't Tell | Yes | Yes | Can't Tell | Yes | Positives: Clear research question in a challenging setting and delivered by non-mental health professionals  Negatives/ Unsures: Unclear about power calculation, small sample |

| **RCTs** | **Research question** | **Random allocation** | **Whether all participants were accounted for** | **Blinding of participants** | **Blinding of investigator** | **Blinding of assessor** | **Whether study groups were comparable at baseline** | **Were groups treated equally** | **Reporting of intervention effects** | **Reporting of the precision of the treatment effects** | **If treatment benefit outweighs the costs** | **Applicability of results to local contexts** | **Value of the experimental condition compared to existing interventions** | **Positives, Negatives or Unknowns** |
| --- | --- | --- | --- | --- | --- | --- | --- | --- | --- | --- | --- | --- | --- | --- |
| Orang et al. (2018) | Yes | Yes | No. Not ITT analysis | No | Can't tell | Yes | Can’t tell. There were some differences but unsure how critical these were (education and perceived stress). | Yes | No. Modified Adverse childhood experience scale and life events checklist were misplaced as secondary outcome (potentially screening measures) as not reported in Results. | No. No confidence interval reported | Can’t tell. But no cost-effectiveness information. | Yes. | Yes | Unknowns:  RCT but there was no power calculation. Sample size was quite small. Same therapists delivered the intervention and treatment as usual. Validated versions of the study measures in the present population were not available. Will be helpful to discuss cultural context of Iran. |
| Dinmohammadi et al. (2021) | Yes | Yes | Yes. | No | Can't tell | Yes | Yes | No. No protocol identified. | No, only *p* value reported. | No | Yes | Yes. Will be useful to discuss cultural context of Iran to see if it can be applied to other contexts. | Yes | Unknowns: RCT. No power calculation  May need to consider ethical issues of not providing intervention to control group.  Unwanted pregnancy seems to be a significant different baseline characteristic between intervention and control. Will be helpful to discuss cultural context of Iran. |
| Bass et al (2014)/ Kaysen et al. (2020)/ Bass et al. (2023) | Yes | Yes | No. Different symptom scores at baseline. | No | No | No | No - similar in clinical characteristics but those in  the individual-support group were younger and  less likely to be married, and they lived with fewer  people | No. Control group receives individual support - the support varies based on psychosocial assistants. Protocol was available online. | Can’t tell. Possible biases: recruitment that resulted in higher average symptom scores in villages that provided individual support because psychosocial assistants recruiting patients knew ahead of time whether they would be providing therapy or individual support. | Yes | Can’t tell. No information on cost-effectiveness. | Yes | Yes | Negatives/ Unknowns/ Source of biases:  Insufficiently powered;  the mental health symptom scale that was used as the primary outcome was not a validated scale; the measure of setting-level insecurity was subjective based on supervisor’s perceptions of incidents of insecurity rather than incident reports/ participants’ perceptions; using visual inspection of slope to determine that sites with higher security level had a faster slope and hence possible more rapid reduction in mental health symptoms may not be a sufficient statistical technique. |
| Knaevelsrud et al (2015) | Yes | Yes | Yes | No | Can’t tell | Can't tell | Yes- except for 'disclosure' | Yes | Yes | No | Can’t tell. No information on cost-effectiveness. | Yes. There was extensive discussion on adaptation to ongoing insecurity context. | Yes | Negatives/ Unknowns: No power calculation.  High drop out |
| Bolton et al (2007) | Yes | Yes | Yes | No | Can’t tell | Yes | Yes - except for age | Yes | Yes | Yes | Can’t tell. No mention of harm, or information on cost-effectiveness. | Yes | Yes | Positives: Include symptoms of Locally Described Syndromes  Negatives:  Same facilitators running both group conditions; idiosyncratic measure |
| Miller et al. (2020) | Yes | Yes | Yes | No | Yes | Yes | Yes | Yes | Yes. Given it's a feasibility study | Yes. Given it's a feasibility study | Can't tell | Can't tell (feasibility study) | Can't tell (feasibility study) | Positives: Implementation fidelity was excellent.  Negatives/ Unknowns: Under-powered as a feasibility study |
| Berger et al. 2007 | Yes | Yes - cluster randomisation by classroom | Yes | No | No - cannot be blinded | Yes | Can't tell - only descriptives presented but no statistical comparisons done for baseline differences | Yes | Yes | No | Can’t tell | Yes | Can't tell | Unknowns: Unsure about power calculation, did not consider baseline difference |
| Tol et al. 2009 | Yes | Yes - cluster randomisation | Yes - ITT analysis | No | No | No | No - Similar level of clinical symptoms but different in demographics e.g. age, gender, displaced, parent-report aggression | Yes - waitlist control, same follow-up period | Yes | Yes | Can't tell due to no reporting of cost effectiveness | Yes | Yes | Positives: Large sample size, RCT, decent follow-up period. included collateral (parent) report.  Negatives: Some of the instruments had less than satisfactory internal reliability |
| Jordans et al., 2010 | Yes | Yes | Can't Tell | No | No | No | No | Chi-square tests showed significant group differences on gender, education, caste/ethnicity, religion and place of residence at baseline. | Can't Tell | Yes | Yes | Can't tell | Can't tell | Positives: Clear stat analysis and CI reported. Overcame many problems with setting challenges- resource |
| Berger et al., 2012 | Yes | Yes | Yes | No | No | Yes | Yes | Yes | Yes | Yes | Yes | Can't tell | Can't tell | Positives: Clear stat analysis and CI reported.  Unknowns: no power calculation |

## **Supplementary Table S2. Study Characteristics.**

| Authors, Year, Country | World Bank Income Group at the time of study (not publication date) | Study Characteristics and Design | Participant Characteristics | Sample Size, Gender & Age |
| --- | --- | --- | --- | --- |
| Abdulah & Abdulla, 2019, Sinjar, Northern Iraq | Lower-Middle Income | Group art-based intervention with pre/post evaluation and no control group. | Yezidi women and girls, survivors of ISIS captivity or invasion. Mostly were single and living with families. | N =14.  Mean Age=19.21 years (*SD* = 4.82). Age range: 10 to 27 years. |
| Ager et al., 2011, Uganda | Low Income | Quasi-experimental design with a comparison group (pre and post assessments). Random quota sampling for intervention schools. 12 months follow-up. | Primary school students who were experiencing ongoing stress and conflict. | N = 403.  Intervention (n= 203);  Comparison (n= 200).  Girls: 202. Boys: 201.  Mean age=10.23 (*SD*=1.61).  Age range: 7 to 12 years. |
| Bass et al., 2013, Democratic Republic of Congo | Low Income | Randomized, controlled, stratified, multi-site trial of Cognitive Processing Therapy for Survivors of Sexual Violence. | Female survivors of sexual violence. Despite regional instability, 80% were living in their territory of origin. 59% of the therapy group were married compared with 43% in the individual support group. Education levels were slightly higher in the individual-support group. | N = 405.  Therapy group (n =157);  Individual support group (n = 248) Women only  Mean age therapy group= 36.9 (*SD*=3.4).  Mean age individual support group- 33.8 (*SD*=12.4).  Age range: not reported. |
| Bass et al., 2022, Eastern Democratic Republic of Congo | Low income | Longitudinal follow-up study on the maintenance of intervention effects of the Bass et al., 2013 Randomized Controlled Trial. | Adult female survivors of sexual violence. Most lived in multi-person households with an average of 4 children, 56% were married, and 27% widowed. 17% experienced sexual violence and 45% witnessed sexual violence in the past 6 months. | N =103 (77% of original treatment group).  Mean age=43.6 (*SD*=12.7).  Age range: 30 to 57 years. |
| Berger et al., 2007, Israel | High income | School-based intervention for elementary-students' terror-related distress; quasi-randomized controlled trial with a waiting list control group. | Elementary school students in Hadera; 7.8% met criteria for PTSD and majority of the students had some PTSD symptoms. | N = 142.  Intervention (n = 70);  Waiting list control (n= 72).  Female: 65; Male: 77.  Mean age and *SD:* not reported.  Age range: 7 to 11 years. |
| Berger et al., 2012, Sderot, South of Israel | High income | Quasi-randomized controlled study of a teacher-delivered intervention for adolescents exposed to ongoing war-related traumatic stress. | Seventh and eighth grade students from a Jewish religious school. Most students were from a lower-middle socioeconomic status and had been exposed to rocket attacks. | N=154.  Girls: 83; Boys: 71.  Mean age= 12.8 (*SD*=1.0).  Age range: 11-13 years. |
| Bolton et al., 2007, Northern Uganda | Low income | Randomized Controlled Trial; multi-site; wait-list control; interventions for depression among adolescent war survivors. | Internally displaced adolescents (Acholi ethnicity). 67% enrolled in school. 42% had a history of Lord's Resistance Army abduction. High level of emotional problems. | N = 314.  Group Interpersonal Psychotherapy: n= 105.  Creative Play (n=105)  Wait-Control Group (n=104).  Female: 180; Male: 134.  Mean age Group Interpersonal Psychotherapy=15.0 (*SD*=1.1).  Mean age Creative Play=14.7 (*SD*=1.0).  Mean age Wait-List Controls=15.2 (*SD*=1.2).  Age range: 14-17 years. |
| Dinmohammadi et al., 2021, Zanjan, Iran | Upper middle income | Randomized controlled trial of solution-focused counselling for pregnant women at risk of domestic violence. | Pregnant women (at or under 27 weeks' gestation) experiencing minor and medium levels of domestic violence. Married for at least one year and living with their spouse. | N =90.  Intervention (n= 45).  Control (n= 45).  Mean age intervention=27.55 (*SD*=5.12).  Mean age control= 27.26 (*SD*=4.46)  Age range: not reported. |
| Farchi & Gidron, 2010  Israel | High income | Randomized controlled trial Focused on mental resilience under war stress. | Adults over 18.  Between 2/3 and 3/4 of the sample were women or working people. Many had experienced a missile hitting their house or had a security room. | N = 64.  Psychological Inoculation (n=33). Ventilation (n=31).  Gender breakdown is not reported. The majority were female.  Age not reported. |
| Gelkopf & Berger, 2009, Israel | High income | Quasi-randomized controlled trial of a school-based, teacher-mediated prevention program for terror-related trauma. | The participants were 7ths and 8th grade male Israeli students from religious backgrounds, of whom 25.3% reported personal exposure to a terrorist attack. | N =114.  ERASE-Stress (n=58).  Waiting List (n= 49).  All males.  Mean age=13.05 (*SD*=0.65).  Age range: 12-14.5. |
| Jordans et al., 2010,  Nepal | Low income | Cluster randomized controlled trial of a classroom-based psychosocial intervention. | Children affected by conflict. Most were Hindu (91%) and lived in their native village (97%). The level of education ranged from grades 2 to 8. | N = 325.  Boys: 167 (51.4%). Girls: 158 (48.6%).  Mean age=12.7 (*SD=*1.04.  Age range: 11-14. |
| Knaevelsrud et al., 2015,  Iraq | The status changed during recruitment to publication  from Lower Middle income to Upper Middle | Randomized Controlled Trial of a Web-based psychotherapy for PTSD. | Arabic-speaking adults with war-related PTSD. 30.2% of participants were married, 25.8% had completed secondary school, and 62% held a university degree. Trauma exposure included sexual violence (39.6%), killing of a family member or close person (15.1%), and exposure to violence, war, or torture (18.9%). | N=159.  Female: 114. Male: 45.  Mean age=28.1 (*SD*=7.43).  Age range: 18-56 years. |
| Miller et al., 2020, Lebanon | Upper middle income but changed to Lower Middle since 2020 | Pilot randomized controlled trial of a caregiver support intervention (CSI) with waitlist control group. | Families displaced by armed conflict. The majority were Syrian refugees (87%), living in poverty in Tripoli, North Lebanon. | N Total=151 parents from 78 families. CSI N= 78, Waitlist N= 73. Female: 79. Male: 72.  Age not reported.  Age range of children: 3-12. |
| Mpande et al., 2013,  Zimbabwe | Low income | Quasi-experimental, pretest/posttest (2 months after) intact groups design; naturalistic setting. | Torture survivors living in situations of continuous traumatic stress and violence. The vast majority of the group, 95%, reported that their families had been threatened, and 36% had family members murdered. The individuals themselves also faced significant threats, with 84% reporting personal threats, 83% being displaced from their homes, and 81% reporting torture. First language chiShona speakers, small-scale farmers, predominantly Christian. | N =146.  Males: 80, Females: 59.  Mean age= 49.5 (*SD*=16.3).  Age range: 19 to 83 years. |
| Orang et al., 2018, Iran | Upper middle income | Randomized controlled trial of Narrative Exposure Therapy (NET) for women exposed to ongoing intimate partner violence. | Iranian women in Tehran experiencing ongoing intimate partner violence and as a result, were diagnosed with PTSD. Most were married and living with a violent partner, with varying education, employment, and financial independence. Participation was often kept secret from husbands. | N = 45  NET (n = 24).  TAU (n = 21).  Mean age NET group= 38.04 (*SD*=9.69)  Mean age TAU=37.28 (*SD*=7.92)  Age range: 16 to 60. |
| Puvimanasinghe & Price, 2016, Sri Lanka | Lower middle income | Randomized controlled trial with a waitlist control group. | Sri Lankan survivors of torture and ill-treatment who sought legal redress and presented with trauma related symptoms. All were over 18, Buddhist, and speak Sinhala. | N= 26.  Males: 16, Females: 10.  Mean age= 40.5 (*SD*=13.26).  Age range: not reported. |
| Tol et al., 2008, Poso, Central Sulawesi, Indonesia | Lower middle income | Cluster randomized trial with wait list control group.  School based mental health intervention. | Primary school children affected by political violence.  Diverse religious background. | N =495.  Boys: 207. Girls: 196.  Mean age= 9.9 (*SD*=1.3) years.  Age range: 7 to 15. |
| Wagner et al., 2012,  Iraq | Lower middle income | Uncontrolled pilot study.  Internet-based intervention for posttraumatic stress disorder. | Participants had experienced an average of 4.5 types of traumatic events. Many had family members kidnapped or killed. 60% completed high school and 27% university. | N = 15.  Females: 13. Males: 2.  Mean age = 29.3 (*SD* = 7.1).  Age range: 20 to 47. |

## **Supplementary Table S3. Conceptualisations of Ongoing Threat.**

**Intimate Partner Violence and Sexual Violence**

| **Authors, Year, Country** | **Conceptualization of Ongoing Threat (as defined by the study or interpreted by the researchers)** |
| --- | --- |
| Abdulah & Abdulla, 2019 Iraq | Yezidi women and girls living in refugee camp who have been systematically targeted by ISIS fighters. Yazidi women have been experienced abduction, rape and being sold or gifted to other fighters. Broader instability in Sinjar at that time. |
| Bass et al., 2013, 2022 Democratic Republic of Congo | The survivors of sexual violence were living in regions affected by conflict and security incidents, including attacks, displacement due to fighting, and robbery by armed groups. These incidents occurred during the trial period, indicating a persistent and ongoing threat environment. Despite this regional instability 80% of the women were living in their territory of origin. |
| Dinmohammadi et al., 2021 Iran | Domestic violence (DV) during pregnancy. While the article focuses on women "at risk of DV" rather than explicitly stating "ongoing risk," the nature of domestic violence and the intervention imply a continuous threat. DV includes physical, psychological, and sexual violence, measured by the Conflict Tactics Scale (CTS-2), with psychological violence being the most observed. |
| Orang et al., 2018 Iran | Intimate Partner Violence (IPV), which included physical, emotional, and psychological abuse. It was defined by the participants' experiences of living in a context of continuous IPV, with ongoing fear of retaliation, lack of legal protection, and active interruptions by IPV occurrences during the study. |

**Conflicts**

| Ager et al., 2011 Uganda | The psychosocial stress and trauma experienced by children due to the aftermath of the conflict in northern Uganda. While the active conflict may have ended, its ongoing impact is defined by widespread psychosocial problems, history of abduction and violence, and the disruption of education and social development for a generation. |
| --- | --- |
| Berger et al., 2007 Israel | Ongoing threat of terrorism in a country affected by war and terrorism. |
| Bolton et al., 2007 Northern Uganda | Ongoing insecurity and internally displacement persons (IDP) stressors in a war-affected country. 42% of the adolescents who participated had been abducted, and most had lived in IDP camps for an average of 5.2 years, reflecting ongoing instability. |
| Farchi & Gidron, 2010 Israel | Continuous war and terrorist attacks related stress caused by nearly daily missile attacks. |
| Gelkopf & Berger, 2009 Israel | Continuous threat of terrorist attacks due to the Israeli-Palestinian conflict. |
| Jordans et al., 2010 Nepal | Ongoing political violence stemming from a 10-year civil war. Although the war had ended, ongoing violence and its socioeconomic impact in Nepal continued to cause lasting psychosocial problems. |
| Knaevelsrud et al., 2015 Iraq | Unstable and insecure setting due to war, ongoing conflict and severe human rights violations that the participants experienced during the study. |
| Miller et al., 2020  Lebanon | Ongoing stressors from displacement by armed conflict and the precarious living conditions faced by Syrian refugees. |
| Mpande et al., 2013 Zimbabwe | Torture survivors living in conditions of continuous traumatic stress due to ongoing political violence (high rates of threats to families, murders of family members, personal threats, displacement, and torture experienced by participants). |
|  |  |
| Puvimanasinghe & Price, 2016  Sri Lanka | Survivors of torture and ill-treatment facing ongoing instability, with persistent fears of reprisal, loss of livelihood, and social stigma related to mental health problems. |
| Tol et al., 2008 Indonesia | Children being exposed to conflict and political instability. |
| Wagner et al., 2012 Iraq | Ongoing violence and economic insecurity. |

**Supplementary Table S4. Intervention details.**

| Authors, Year, Country | Intervention Details (Content, Frequency, Length) | International Red Cross and Red Crescent Movement’s Mental Health and Psychosocial Support Framework | Mode of Delivery (Setting/Where; Deliverer) | Tailoring and Modifications (Planned; During Study) | How Well (Fidelity & Adherence Assessment) |
| --- | --- | --- | --- | --- | --- |
| **Abdulah & Abdulla, 2019**   Sinjar, Northern Iraq | Two-month art-based programme with four sessions per week (4 hours each).  Training in painting/drawing to express perspectives on life and nature. The art instructor taught techniques (shapes, shading), encouraged peaceful, non-violent themes, and explained colour symbolism. A public exhibition was held at Sharya IDP Camp, during which participants showcased and discussed their artworks.  A psychiatrist offered mental health recovery sessions to 4 survivors of captivity. | Focused psychosocial support (the art groups were delivered by artist) | Face-to-face group art sessions delivered by an art instructor.  A psychiatrist offered individual support (for some participants)  Setting: Sharya Internally Displaced Persons (IDP) Camp. | None reported. | Fidelity and adherence were not reported. |
| **Ager et al., 2011**  Uganda | Psychosocial Structured Activities (PSSA) program: 15 class sessions over five weeks designed to increase children's resilience through structured activities involving drama, movement, music, and art.  Other components included community service and parental engagement. | Focused Psychosocial Support | School-based, Group.  Delivered by trained school teachers.  Save the Children in Uganda (SCiUg) Child Resilience Project staff delivered the parental engagement and supervisory visits. | None mentioned/ reported.  Challenges in implementation due to resource constraints and a lack of supervision. | None reported.  Teachers received training, but supervision was limited. |
| **Bass et al., 2013** ; **Bass et al. (2022) (long-term follow-up)** Democratic Republic of Congo | Group Cognitive Processing Therapy (1 individual session and 11 group sessions).  OR Individual psycho-social support as desired, including economic, medical, and legal referrals. | Psychological Support (Group Cognitive Processing Therapy arm)  and  Focused Psychosocial Support (control) | Face-to-face.  Delivered by psychosocial assistants who completed two weeks of in-person training with US-based trainers. | **Content tailoring:** Therapy adapted for illiterate participants and those potentially exposed to ongoing violence.  Adaptations included simplification of materials for understanding.  **Cultural competence**: Standard CPT model modified to fit Congolese culture, paraprofessional delivery, adaptations for beliefs about sexual assault's impact on social status (e.g., local "stuck points" examples)  **Context adaptation:** PSAs sometimes organized meetings in forest/fields during displacement to maintain therapy access. | Ongoing supervision was provided through a multitiered system, including weekly telephone or in-person meetings with Congolese psychosocial supervisors and a bilingual clinical social worker.  Fidelity/Adherence to the protocol was assessed using checklists of key treatment elements and global ratings of treatment knowledge and skills, observed by supervisors during group sessions. |
| **Berger et al., 2007**  Israel | A School-Based Program.  Duration: Eight 90-minute sessions.  Content: Homework review, warm-up, experiential group activity, psychoeducational presentation, practical coping skills training, closure. | Focused Psychosocial Support | Face-to-face group  Delivered by trained teachers. | None mentioned/ reported. | Trainers offered three 3-hour supervisory sessions with teachers. Monitored through point-by-point discussions of training procedure.  Fidelity and adherence assessment were not reported. |
| **Berger et al., 2012**   Sderot, South of Israel | ERASE-Stress school-based intervention.  The intervention placed less emphasis on traumatic memories and focused more on developing coping strategies for daily stressors; recognising and managing emotions such as anger, grief, fear.  Duration: 16 weekly, 90-minute classroom sessions. | Focused Psychological Support | Face-to-face classroom group. Delivered by teachers. | **Content cultural integration**: Two major adaptations from original ERASE-Stress: Emphasized religious/spiritual practices; as threat was ongoing, added affect modulation, self-affirmations, practice combating fears, and more social skills. | Trainers observed teachers' implementation. Observations were held for each of 16 sessions. Ratings on a 6-point Likert-type scale, with all scores 4 or 5, indicating high fidelity. |
| **Bolton et al., 2007**   Northern Uganda | Group Interpersonal Psychotherapy (IPT-G) adapted for adolescents.  OR Creative Play (CP): Activity-based interventions serving specific psychosocial goals (e.g. building trust) selected from the War Child Holland manual developed for war-affected youth.  Duration: both comprised 16 weekly meetings, 1.5 to 2 hours each. | Focused Psychological Support | Face-to-face groups delivered by trained lay facilitators.    One or two individual meetings preceded the group sessions.  Setting: camps for internally displaced persons in northern Uganda. | **Relationship fidelity:** A treatment manual outlining IPT-G strategies and techniques was adapted for local use. Preliminary qualitative work in northern Uganda indicated that its emphasis on interpersonal triggers and group relationship building was culturally compatible. | Facilitators received weekly supervision from experienced local staff who had IPT-G experience, who in turn were supervised weekly by a US-based trainer.  Weekly written reports were reviewed to monitor adherence. |
| **Dinmohammadi et al., 2021**   Zanjan, Iran | **Solution-focused counselling:** Six weekly 90-minute sessions. | Psychological Support | Face-to-face individual counselling delivered by specialists.  Setting: in an urban health care centre. | None reported | Fidelity and adherence were not reported. |
| **Farchi & Gidron, 2010**   Israel | Ventilation: participants were invited to share their views on security, describe their coping strategies, and indicate what helps them cope the most. Emotional expression and coping efforts were encouraged.  Psychological Inoculation (PI): participants were asked to refute "challenging sentences" related to stress, coping self-efficacy, and PTSD symptoms  Duration: two phone sessions, one week apart. | Psychological Support | Individual sessions conducted over the phone and delivered by counsellors. | None reported | Fidelity and adherence were not reported. |
| **Gelkopf & Berger, 2009**  Israel | ERASE-Stress intervention: Twelve 90-minute weekly sessions.  Psychoeducation around coping, emotional awareness, managing emotions including fear, grief, anger, boosting self-esteem. | Focused Psychological Support | Face-to-face classroom group.  Delivered by teachers | None reported. | Trainers observed the sessions and used a 6-point Likert scale to evaluate adherence in five areas: adherence to topics, exercises, active participation, homework discussion, and overall course orientation.  Teachers attended three 90-minute supervision sessions with the author of the manual to ensure consistency. |
| **Jordans et al., 2010**   Nepal | Classroom-Based Intervention (CBI): 5-week, 15-session (approx. 60-minute each) protocolized group intervention. Eclectic, based on creative-expressive therapy, play, and CBT. Combined psychoeducation, socio-drama, movement/dance, group cohesion activities, stress inoculation, and trauma-processing via (voluntary) narrative exposure through drawings. | Focused Psychological Support (with trauma exposure component) | Classroom-based, Group, Face-to-face  Delivered by: Four local research assistants who received 3 weeks training).  Regular supervision by an experienced counsellor. | None reported | Fidelity and adherence were not reported.  Facilitators received training and ongoing supervision. |
| **Knaevelsrud et al., 2015**  Iraq | Web-Based Psychotherapy for Posttraumatic Stress Disorder (CBT for PTSD):  Content: 2 weekly 45-minute cognitive behavioural interventions via Internet over a 5-week period (10 sessions total). 10 writing assignments.  Components include writing the trauma narrative, cognitive restructuring. | Psychological Support | Online, Individual CBT. Delivered by eight native Arabic-speaking psychotherapists or psychiatrists living in Iraq, Palestine, Syria, the Emirates, or Europe. | A Dutch Internet-based CBT manual was translated into Arabic and culturally adapted. It encouraged a more directive therapeutic stance, incorporating quotes/metaphors from the Koran, and respecting cultural norms regarding family and discouraging disclosure of sexual violence to family members due to potential serious social consequences (dishonour). | Fidelity and adherence were not reported.    Therapists received 7-day training and ongoing supervision. |
| **Miller et al., 2020**   Lebanon | Caregiver Support Intervention (CSI): Nine-session group intervention. Offered separately to women and men (co-facilitated by same-gender facilitators).  Content included stress and relaxation, anger management, grounding, parenting stress, positive parenting | Focused Psychosocial Support | Group, Face-to-face  Delivered by: Trained non-mental health specialists (six days in-class training, three on-site observations with feedback, weekly supervision with trained social worker supervised by the principal investigator and local Lebanese psychologist). | **Relationship fidelity:** The intervention went through several iterations of implementation and revision prior the RCT based on feedback from participants and facilitators. | The field supervisor conducted three observational visits to each CSI group using a fidelity checklist.  All activities were delivered as designed in six of seven groups; in one men’s group, facilitators initially missed two activities per session due to insufficient preparation. |
| **Mpande et al., 2013**  Zimbabwe | The Tree of Life Trauma Healing Workshop (TOL): draws on ecological systems theory and shares features with exposure-based CBT and testimony therapy.  Duration: 3 days, comprising eight guided conversations ("circles") where participants reflected on the childhood, wider systems, ancestry, connections, difficult experiences. | Psychological Support | Group, Face-to-face.  Delivered by facilitators (qualification unknown). | None reported | Fidelity and adherence were not reported.  Close supervision of facilitators was offered. |
| **Orang et al., 2018**  Iran | Narrative Exposure Therapy (NET).  Therapy lasted between 3 and 6 months, with variability due to cancellations and interruptions. Participants completed between 8 and 15 sessions across treatment groups, with a mean of 11.38 sessions (SD = 1.55). | Psychological Support | Individual, Face-to-face.  Therapy was delivered by specialist therapists with master’s-level psychology qualifications, under the supervision of a doctoral-level psychologist.  Sessions were delivered in community settings close to participants’ homes. | **Context adaptations:** Because participants experienced continuous threat and violence from their partners, one to two sessions were allocated to address safety concerns and current violence throughout the entire process of NET.  Interviews and treatment were organised with flexible logistics tailored to each participant’s needs to keep the participation secret from the family. | The initial NET sessions were supervised by a clinical psychologist with expertise in NET, alongside a local NET expert to ensure adherence to the NET manual guidelines. |
| **Puvimanasinghe & Price, 2016**   Sri Lanka | Testimonial Therapy (TT): focused on the narration of the survivors' traumatic experiences over five sessions. Components included audio-taped testimony and presented with significant others. | Psychological Support | Individual, Face-to-face  Delivered by Trained non-specialists | A therapy manual was adapted for the Sri Lankan context and designed for trained non-specialists. | Fidelity and adherence were not reported. |
| **Tol et al., 2008**  Poso, Indonesia | Classroom-based intervention: 15 sessions with groups of about 15 children, over 5 weeks, using a manualized approach. Integrates CBT techniques with cooperative play and creative-expressive exercises (drama, dance, music) in a structured phased program. Included trauma narrative through art and drama, as well as stabilisation. | Focused Psychological Support | Group, Face-to-face  Delivered by locally trained paraprofessionals (no formal mental health training, but humanitarian volunteer experience). | None reported | Fidelity/Adherence:  Assessed by multiple independent research assessors scoring 14 videotapes of randomly selected sessions using a structured checklist. Average treatment adherence was 89.76%. Interventionists did not participate in assessments. |
| **Wagner et al., 2012**   Iraq | Internet-based (trauma-focused) Cognitive-Behavioural Treatment. Planned Duration: 5 weeks. Actual Average Duration: 12 weeks (range 6-25 weeks).  Content: Three treatment phases: Self-confrontation (describing traumatic event); Cognitive reappraisal; Social sharing. Participants were given two weekly 45-minute writing assignments over 5 weeks. | Psychological Support | Online, Individual.  Delivered by: Arabic-speaking psychotherapists or psychiatrists living in Iraq or neighbouring countries. | The manual was translated into Arabic and culturally adapted for the Iraqi population. | Fidelity and adherence were not reported.  Training and supervision were provided to therapists. |

**Supplementary Table S5. Outcomes and Implementation Challenges/Considerations**

| Authors, Year, Country | Outcome Measures | Findings/Outcomes | Limitations, Implementation Challenges/Considerations) |
| --- | --- | --- | --- |
| **Abdulah & Abdulla, 2019**  Northern Iraq | **Flourishing Scale (FS):**  An 8-item measure assessing socio-psychological well-being, including positive and negative relationships, sense of purpose, engagement in activities, and perceived competence. Scores ranged from 8 (lowest well-being) to 56 (highest well-being), with higher scores indicating greater psychological flourishing. | Overall psychological wellbeing improved among both survivors of captivity (n=4) and other participants (n= 10), *p* < 0.1, *d* = 1.89 | Small sample size (14 participants) due to only one artist facilitating the intervention;  Lack of standardisation or training in clinical art therapy in Iraq. |
| **Ager et al., 2011**  Uganda | **Child well-being:** Composite scale score (6-60) as reported by children, parents, and teachers.  The scale was derived through participatory focus groups (derived specifically for this study). | Children who received the intervention showed significantly greater improvements in well-being than those in the comparison group, based on reports from children (*B* = 5.4, *SE* = 1.1, *p* < .001) and parents and (*B* = 4.0, *SE* = 1.5, *p* = .01, respectively), but not from teachers (*B* = 2.0, *SE* = 1.2, *p* > .1). | Significant loss of participants at follow-up due to return migration (150 participants lost). The retained sample was older and reported higher well-being at baseline;    No standardised measure of well-being;  The teacher deployment policy led to different teachers reporting on the child's well-being at different data-collection points. |
| **Bass et al., 2013**  Democratic Republic of Congo | **Functional impairment:** Assessed based on how difficult it was to perform 20 key daily tasks identified from qualitative data. A 5-point Likert scale was used (0=no difficulty to 4=often unable). Higher scores indicated greater impairment. | The therapy group showed greater improvements on functional impairment scores than the individual support group at the end of treatment and 6 months later, *p* < 0.1, *d* = 1.1. | Baseline differences in symptom severity between study groups limit comparability.  Randomization limitations from grouping two to four nearby villages by language and proximity, but the assumption of similarity was not empirically tested;  Possible recruitment biases. Psychosocial assistants knew allocation during recruitment, which may have influenced the process;  Group therapy arm received more supervision. |
| **Bass et al., 2022**  Democratic Republic of Congo | **Functional impairment:**  Daily functioning measure as described above. | Improvement in functional impairment at the end of treatment (*MD*= −0.94, *SE*= 0.12, *p* <0.0001), 6 months after end of treatment (*MD*= −0.93, *SE*= 0.09, *p* <0.0001, 18 months after end of treatment (*MD*=−0.73, *SE*= 0.11, *p* <0.0001) and Long-term follow-up (*MD*= −0.47, *SE*= 0.09, *p*<0.0001). | Uncontrolled study; Unmeasured cumulative trauma exposure. |
| **Berger et al., 2007**  Israel | **Functional impairment:** Measured using four items from the Child Diagnostic Interview Schedule (social relationships, school performance, family relationships, after-school activities). The functional impairment score was the sum of items. Higher scores indicated greater impairment. | Significant improvements in functional impairment two months post treatment (Time X Group interaction, *F* (1,140)= 40.59, *p* < .001) compared to waiting list control.  There was a larger improvement in functional impairment in boys (*F* (1,138)= 4.16, *p* <.05). | Low parental consent rate (46.6%) may introduce sample bias;  Not able to isolate intervention-specific impact due to waiting list control group. |
| **Berger et al., 2012**  Israel | **Functional impairment:** Measured using four items from the Child Diagnostic Interview Schedule (social relationships, school performance, family relationships, after-school activities). The functional impairment score was the sum of items. Higher scores indicated greater impairment. | Significant improvements in functional impairment compared to waiting list controls (Time X Group interaction, *F* (1,152) = 20.34, *p* < .001), medium effect size, *η²*=0.12). | Low parental consent rate may introduce sample bias. |
| **Bolton et al., 2007**  Uganda | **Functional impairment:** A local function measure was developed based on qualitative data, with two gender-specific scales reflecting important activities for youth in camps.  Overall score for girls: 0-36 (9 activities) and 0-20 for boys (5 activities).  Higher scores indicated greater impairment. | IPT-G participants reported greater reductions in functional impairment scores, but the overall differences compared with controls did not reach statistical significance (*p =* .69 for girls, *p = .2* for boys). | The functional impairment measure was derived from qualitative data, yielding a limited number of items focused on task-based functioning rather than the broader social and interpersonal domains targeted by the interventions;  The study was not powered initially to detect gender-specific differences. |
| **Dinmohammadi et al., 2021**  Zanjan, Iran | **Quality of life:**  Short Form Health Survey (SF-36): Evaluates quality of life in two dimensions: physical and psychological health. | The intervention group showed a statistically significant improvement in quality of life compared to the control group (*p*= 0.001). Nonparametric ANCOVA values and effect sizes were not reported. | Small sample;  Self-reported questionnaires may have been influenced by participants’ reluctance to disclose personal issues due to fear of revealing their private life issues. |
| **Farchi & Gidron, 2010**  Israel | **Daily functioning:** Assessed by a 1-item scale, asking "To what extent did you succeed to carry out what you planned during the last week?" (1=not at all; 10=succeeded to do everything).  To minimize participant burden during the war, only very brief measures were used for assessment. | No significant time X group interaction improvements in daily function were found, *F*(1,64)= 1.15, *p*=0.29 | Significant between-group baseline differences in daily functioning for ventilation group. |
| **Gelkopf & Berger, 2009**  Israel | **Functional impairment questionnaire:** 7 items from the DISC Predictive Scales (DPS) derived from the Child Diagnostic Interview Schedule covering social relationships, school performance, family relationships, and after-school activities. 5-point scale (1=not at all to 5=very much).  Satisfactory internal reliability (Cronbach's Alpha = .78).  Scores were summed, with PTSD functional impairment criteria met if participants reported ‘much’ or ‘very much’ impairment in at least one domain. | Functional problems improved significantly compared to the waiting-list control group (Time X Group interaction, *F* (1,106)= 15.50, *p* < .001). No effect sizes reported. | The intervention was compared with the waiting list, not with another active intervention. |
| **Jordans et al., 2010**  Nepal | Function impairment was assessed with a 10-item Children's Function Impairment (CFI) questionnaire developed in Nepal. | Functional impairment significantly improved compared to the control group, *p* < 0.01, *d* = .58. | Low internal reliability of some instruments (especially SCARED-5), affecting pre-post comparisons. |
| **Knaevelsrud et al., 2015**   Iraq | **Quality of life:**  Assessed with EUROHIS-QOL eight-item scale. It measured 4 domains of life quality (psychological, physical, social, environmental), each with 2 items. Higher scores indicated better quality of life. | Intervention (web-based trauma-focused CBT) led to a significant increase in QoL life satisfaction, *F* (1,157) =44.20, *p*<.001, *d*= 0.84.  Quality of life improved with moderate within-group effects in intention-to-treat analysis (*d*=0.76) and large effects among completers (*d*=1.30) | High attrition rate (41%) due to ongoing violence, economic insecurity, concerns about neutrality, technical issues, and privacy concerns. |
| **Miller et al., 2020**  Lebanon | **Caregiver Psychosocial Wellbeing:** Warwick-Edinburgh Mental Wellbeing Scale (WEMWBS, 14-item).  **Child Psychosocial Wellbeing-Parent Report:**  Kid-KINDL for Parents (children 7+) and Kiddy-KINDL for Parents (children 3-6). 24 items (4 school items dropped, 4 optional mental health items added), 5 answer choices (never to all the time), total scores 24-120. (Only female caregivers completed Kindl due to cultural norms).  **Child Psychosocial Wellbeing- Child Report:**  Kid-KINDL self-report administered to children aged 7–12 years. | Caregiver psychosocial wellbeing improved significantly (*p* < .01, *95% CI* = 1.35–4.49, *d* = 0.43).  Parent-reported psychosocial wellbeing also showed significant improvement (*p* < .01, *95% CI* = 2.24–9.96, *d* = 0.51), whereas child-reported psychosocial wellbeing showed a non-significant increase (*p* = .27, *95% CI* = −1.91–6.50, *d* = 0.26). | No family observations were conducted to triangulate the findings due to resource constraints and the setting.  Low test-retest reliability of certain measures (Kindl-child self-report) (*ICC*= 0.53) |
| **Mpande et al., 2013**  Zimbabwe, Murewa district | **Zimbabwe Community Life Questionnaire (ZCLQ):** Measured behaviour change reflecting healing through an ethnographic approach. Included scales for Global, Community engagement, Contribution to others' lives, and Attitudes to community healing. | Both interventions demonstrated small to moderate improvements across all ZCLQ scales. Only the effect sizes were reported.  ZCLQ Global showed improvements for Tree of Life (*d* = 0.46) and Psychoeducation (*d =* 0.57). Community Engagement improved for Tree of Life (*d* = 0.44) and Psychoeducation (*d* = 0.39). Contribution to the Lives of Others improved for Tree of Life (*d* = 0.27) and Psychoeducation (*d* = 0.70). Attitudes to Community Healing improved for both Tree of Life and Psychoeducation (*d* = 0.39).  The Tree of Life and Psycho-education were statistically equivalent for all client outcomes (F not reported), except for ZCLQ-Contribution to Lives of Others, where psycho-education participants showed more improvement, *F*(1, 134) =4.30, *p* =.04, *d*=0.29. | Significant baseline group differences.  Interventions may have contained more common components than planned (e.g., spontaneous storytelling in Psychoeducation group). |
| **Orang et al., 2018**  Iran | The Work and Social Adjustment Scale (WSAS): a five-item measure of impairment. Scored on a scale from 0 to 8, 0 indicating no impairment at all and 8 indicating very severe impairment. | Within-group improvements were large for NET (*g* = 0.88 at 3 months and *g* = 0.80 at 6 months), and TAU (*g* = 0.75 at 3 months and *g* = 0.97 at 6 months).  Between-group effect sizes were negligible (*g* = 0.14 at 3 months, *g* = 0.00 at 6 months), indicating that NET and TAU were comparable in improving functioning. | Small sample size |
| **Puvimanasinghe & Price, 2016**   Sri Lanka | Sri Lanka Index of Psychosocial Status (SLIPSS-A):  The SLIPSS-A was specifically developed as a general measure of psychosocial functioning in rural Sri Lankan, Sinhalese-speaking adults affected by trauma.  26-item measure of psychosocial functioning (5-point scale, 0=never to 4=6-7 days/week). Lower scores = better functioning.  World Health Organization Five Well-being Index (WHO-5): Screening tool for mental well-being. | Psychosocial functioning improved over time for all participants (*p*=.02), with survivors receiving testimonial therapy showing greater improvement than controls, *p* = .01, effect size = 0.60.  Emotional well-being (WHO-5) showed nonsignificant change over time or between groups (*p* = .82, effect size = 0.09). | Small sample size.  Possible control group contamination through informal interactions with the treatment group.  Psychometric limitations of outcome measures. |
| **Tol et al., 2008**   Poso, Central Sulawesi, Indonesia | Function impairment:  Impairment in functioning was measured via a contextually constructed 10-item child-rated checklist.    Measured difficulties in activities on a 4-point scale (higher score = more difficulties). | Significant effects of sex on change in functioning (3-way interaction time X intervention X sex, *β* = 2.75; *95% CI =* 1.15 to 4.34). *P* value was not reported.  2-way interaction subgroup analyses (time X intervention) in in subgroups showed that treatment was effective in reducing function impairment for girls, *β* = 1.64; *95% CI* = 0.27 to 3.02. No significant effects of treatment were found among boys. | Unsatisfactory psychometric properties of some scales. |
| **Wagner et al., 2012**   Iraq | Quality of life: Assessed with the EUROHIS-QOL eight-item scale, measuring four domains (psychological, physical, social, environmental), each with two items. Higher scores indicated a better quality of life. | Quality of life (EUROHIS) increased significantly at post-treatment, *p*<0.0001, *d*=1.17. | Pilot study; no randomized control group.  Some patients expressed doubts regarding the neutrality of the website and the treatment provided.  Logistical challenges due to travel restrictions and visa issues for program coordinators and therapists. |
